# Polyamines stimulate the protein synthesis of the translation initiation factor eIF5A2, participating in mRNA decoding, distinct from eIF5A1

**DOI:** 10.1016/j.jbc.2025.110453

**Published:** 2025-07-04

**Authors:** Masato Suzuki, Takehiro Suzuki, Yoshio Nakano, Ken Matsumoto, Hitomi Manaka, Masahiro Komeno, Shoma Tamori, Akira Sato, Naoshi Dohmae, Kazunori Akimoto, Satoru Miyazaki, Takashi Suda, Toshihiko Toida, Keiko Kashiwagi, Kazuei Igarashi, Kyohei Higashi

**Affiliations:** 1 Faculty of Pharmaceutical Sciences, Tokyo University of Science, Noda, Chiba, Japan; 2 RIKEN Center for Sustainable Resource Science, Wako, Saitama, Japan; 3 Cancer Research Institute, Kanazawa University, Kanazawa, Japan; 4 Graduate School of Pharmaceutical Sciences, Chiba University, Chuo-ku, Chiba, Japan; 5 Faculty of Pharmacy, Chiba Institute of Science, Choshi, Chiba, Japan; 6 Amine Pharma Research Institute, Innovation Plaza at Chiba University, Chuo-ku, Chiba, Japan

**Keywords:** polyamine, spermidine, eukaryotic translation initiation 5A (eIF5A), cell proliferation, proteomics

## Abstract

Polyamines are present in all living organisms, and their homeostasis is closely associated with human health and disease. Furthermore, they are small aliphatic cations that exhibit multifunctional activities through interactions with acidic substances, thereby precluding our understanding of their molecular functions in biological processes. eIF5A1 and eIF5A2 share high amino acid sequence similarity, and hypusination, using spermidine, is essential for their functions. eIF5A1 is ubiquitously expressed in all tissues and is essential for normal cell growth, whereas eIF5A2 is often expressed in human cancer tissues; however, the functional differences between eIF5A1 and eIF5A2 remain unclear. Here, we found that eIF5A2 is regulated by polyamines at the translational level and that eIF5A2, rather than eIF5A1, is important for cancer cell growth. The translational initiation of *eIF5A2* mRNA was negatively regulated by miR-6514-5p at the 5′-UTR, and polyamines inhibited this miRNA function, facilitating eIF5A2 synthesis. A proteomic analysis of cells with either eIF5A1 or eIF5A2 silenced showed distinct profiles. In addition, polyamines upregulated the expression of five ribosomal proteins, particularly RPS27A, RPL36A, and RPL22L1, which are associated with cancer malignancy. Our findings reveal an important role for eIF5A2, regulated by polyamines and miR-6514-5p, in cancer cell proliferation, suggesting that the interaction between eIF5A2 and ribosomes, which regulate cancer progression, is a selective target for cancer treatment.

Polyamines (putrescine [PUT], spermidine [SPD], and spermine [SPM]) are present in all living organisms and play important roles in cell growth, viability, and differentiation (1, 2). PUT, SPD, and SPM contain 2, 3, and 4 amino groups, respectively, and they are present at millimolar concentrations intracellularly. PUT is synthesized from ornithine by ornithine decarboxylase (ODC), a rate-limiting enzyme involved in the polyamine biosynthesis pathway. SPD is synthesized from PUT by spermidine synthase, and SPM is synthesized from SPD by spermine synthase. SPD is a substrate for hypusination, which occurs uniquely at a specific lysine residue (Lys^51^ in yeast and Lys^50^ in humans) in the eukaryotic translation initiation factor (eIF)5A. This process is catalyzed by two enzymes, deoxyhypusine synthase (DHS) and deoxyhypusine hydroxylase (DOHH) (3, 4). In 80S ribosomes, hypusinated (hyp)-eIF5A1 is located adjacent to the P-site of the tRNA, overlapping with the E-site. Moreover, its hypusine (*N*^ε^-(4-amino-2-hydroxybutyl)lysine) residue interacts with the CCA-end of P-site tRNA (5, 6), stimulating the translational elongation of polyproline or other difficult-to-translate peptide motifs and translation termination (7, 8). Elevated levels of polyamines, ODC, and eIF5A proteins are often observed in various cancer types and are associated with their hyperproliferative phenotypes (1, 9, 10, 11). Considering that α-difluoromethylornithine (DFMO), an ODC inhibitor, and *N*^1^-guanyl-1,7-diaminoheptane (GC7), a DHS inhibitor (12), can inhibit the growth of cell lines and tumor tissues by impairing polyamine biosynthesis or hypusination, the physiological functions of polyamines, particularly SPD and hyp-eIF5As, have been extensively studied. In most eukaryotes, two *eIF5A* genes encode eIF5A1 and eIF5A2, which share 84% (in humans) and 82% (in mice) amino acid (AA) sequence identity with each other (3, 4). Notably, the homozygous knockout of *eIF5A1* in mice results in embryonic lethality between E3.5 and E7.5 (13); however, the *eIF5A2* gene in mice is not essential for normal development and viability (14). In addition, eIF5A1 is ubiquitously expressed, whereas eIF5A2 is expressed only in the testes and brains of mice (14). Since the homozygous depletion of *Dhs* and *Dohh* in mice results in embryonic lethality (13, 15, 16), studies have focused on the hypusination of eIF5A1 to understand better the physiological functions of polyamines in health and disease. However, eIF5A2 has become increasingly recognized as an oncogene in cancer development and progression (4).

Recently, several studies have reported that SPD supplementation extends the lifespan and health span of organisms such as *Saccharomyces cerevisiae* (BY4741 strain) (17), *Drosophila melanogaster* (W^1118^ strain) (17), *Caenorhabditis elegans* (17), and mice (18), as well as immune cells from mice (17, 19, 20, 21). Furthermore, SPD protects against age-induced memory impairment in *D. melanogaster* (22, 23) and mice (23, 24). Almost all of the anti-senescence effects of SPD depend on the activation of mitochondrial functions through the induction of autophagy-related genes (17, 18, 22, 23, 25). Hyp-eIF5A1 also enhances the translation of the autophagy transcription factor TFEB and autophagy-related protein ATG3 (20, 26). In the presence of oxygen, nonproliferating (differentiated) tissues metabolize glucose to generate pyruvate *via* glycolysis and then completely oxidize most of the pyruvate in the mitochondria to CO_2_ during oxidative phosphorylation (27). In contrast, in cancer cells, most glucose tends to be converted to lactate regardless of the presence of oxygen (aerobic glycolysis) (27), but the polyamine-stimulation mechanism underlying cancer progression, especially the genes modulated by polyamines, remains unclear.

We previously found that polyamines exist primarily as polyamine–RNA complexes (the amount of bound polyamines is 2–6.5 mol/100 mol of RNA phosphate) in *Escherichia coli* (28), bovine lymphocytes, and rat liver (29). In addition, polyamines promote cell growth by facilitating the translation of a specific protein *via* structural changes in mRNA, tRNA, and ribosomal RNAs in *E.coli* (30).

In this study, we found that gene expression (5.3%) modulated by polyamines resulted in the activation of glycolysis, rather than mitochondrial respiration, in HeLa S3 cells. We discovered that eIF5A2, regulated by polyamines, and miR-6514-5p play a major role in cancer cell growth, and the individual genes whose expression was upregulated by eIF5A2 were distinct from those whose expression was upregulated by eIF5A1. Moreover, the expression of five ribosomal proteins, especially RPS27A (31), RPL36A (32, 33), and RPL22L1 (34, 35, 36), which are associated with cancer malignancy, was upregulated by polyamines. Our findings reveal that eIF5A2, regulated by polyamines and miR-6514-5p, participates in the translational machinery that is adapted to cancer progression and that this can be targeted for cancer treatment.

## Results

### Genes modulated by polyamines activate glycolysis rather than mitochondrial respiration

The effect of polyamine depletion on HeLa S3 cell growth was examined after DFMO treatment. After HeLa S3 cells were treated with 5 mM DFMO for 72 h, PUT and SPD contents reached a minimal level, and the cell number decreased to approximately 40% of that in the control on day 3 (Fig. S1). When 25 μM of SPD was added to DFMO-treated cells, it was quickly incorporated by the cells, restoring cell growth. Based on these observations, we used these concentrations of DFMO and SPD to treat HeLa S3 cells for 72 h, and proteomic analysis was performed *via* nano LC-MS/MS.

**Figure 1:**
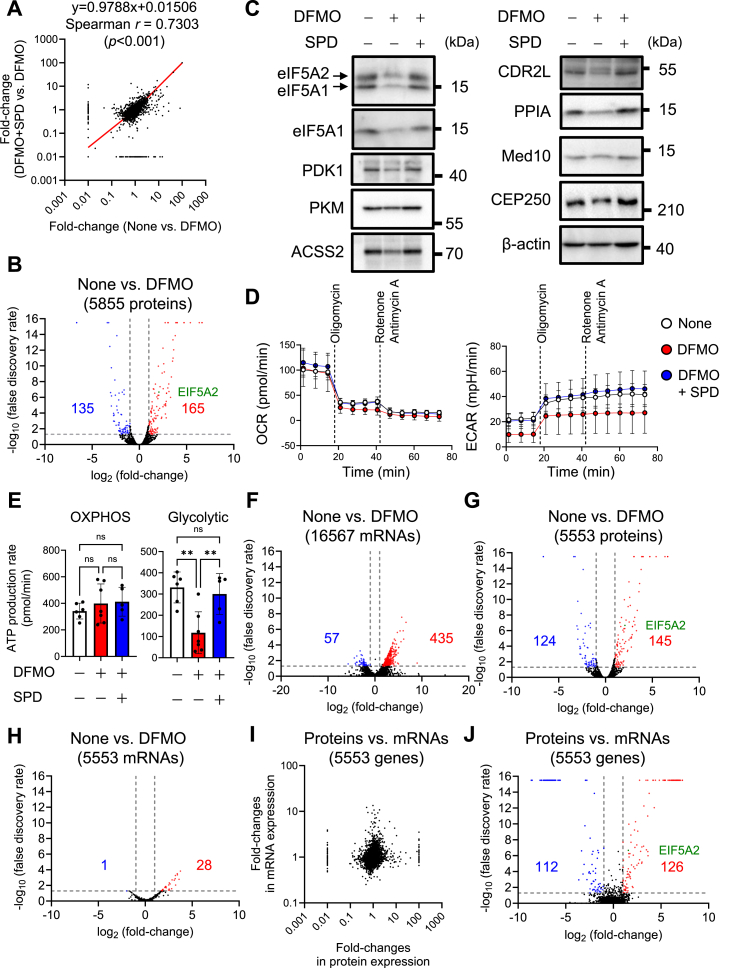
**Altered gene expression mediated by polyamines results in the activation of glycolysis rather than mitochondrial respiration**. *A*, correlation of fold-change between the None vs. α-difluoromethylornithine (DFMO) and DFMO + spermidine (SPD) vs. DFMO groups. *B*, volcano plot of 5855 proteins in the None vs. DFMO comparison. The cut-off values for significant fold-changes, >2.0 or <0.5 (log_2_ values, >1.0, or < −1.0), and *p*-values, <0.05 (−log_10_ value, >1.301) are indicated by broken *gray lines*. *C*, effects of DFMO and SPD on the expression levels of upregulated proteins (>2.0) mediated by polyamines (*n* = 3). 20 micrograms of cell-lysate protein was loaded for Western blot analysis. Experiments were repeated three times, and reproducible results were obtained. *D*, effect of polyamines on oxygen consumption rate (OCR) and extracellular acidification rate (ECAR) in HeLa S3 cells (*n* = 6). OCRs and ECARs of 4.0 × 10^4^ cells/well were measured after culture for 48 h in the presence or absence of 5 mM DFMO and 25 μM SPD. *E*, ATP production rate calculated using OCRs and ECARs. Data are expressed as the mean ± S.D. *(error bars*). ∗∗*p* < 0.01; ns, not significant. *F*, volcano plot of 16,567 RNA ratios in the None vs. DFMO comparison. *G* and *H*, volcano plots of proteins in (*G*) and mRNAs of 5553 genes in (*H*) of the None *versus* DFMO (*n* = 3) comparison. *I*, scatter plots of the comparison between proteins and mRNAs of 5553 genes in the None *versus* DFMO comparison. *J*, volcano plots of the protein/mRNA ratios in the None *versus* DFMO comparison (*n* = 3).

The expression datasets comprising >6700 identified proteins were compiled to generate a list of detailed protein IDs, gene symbols, and fold-changes in non-treated HeLa S3 cells (None) *versus* DFMO-treated cells (DFMO) or DFMO+SPD-treated cells (DFMO+SPD) vs. DFMO (Table S1). Given that a positive correlation was observed in the fold-changes between the None vs. DFMO and DFMO+SPD *versus* DFMO comparisons (Fig. 1*A*), we considered that the side effects of DFMO on gene expression in HeLa S3 cells might be negligible. A volcano plot of the geometric means of fold-changes and combined Stouffer’s *p*-values of the 5585 proteins (except for unnamed proteins) revealed 300 (5.34%) proteins of which expression levels were altered by DFMO treatment (Fig. 1*B*). Among them, expression levels of pyruvate dehydrogenase kinase 1 (PDK1) and pyruvate kinase 2 (PKM2), which are associated with aerobic glycolysis and growth in cancer cells (37, 38), acyl-coenzyme A synthase short-chain family member 2 (ACSS2) (39), and eIF5A2 were upregulated; however, the expression levels of only a few genes related to oxidative phosphorylation (OXPHOS), autophagy, and TCA cycle were modulated (Fig. S2). Results of Gene Ontology (GO) analysis of the 500 most-regulated proteins were consistent with the observation that polyamine depletion influences ER (40) and N-glycosylation (41) (Fig. S3); however, alterations in OXPHOS, autophagy, and the TCA cycle were not observed. Next, we comprehensively determined the fold-changes in the expression levels of detected proteins and found that those of the genes related to glycolysis and autophagy were moderately upregulated, whereas those of OXPHOS genes were downregulated by polyamines (Fig. S4).

Altered eIF5A2, PDK1, PKM2, ACSS2, cerebellar degeneration-related protein 2-like (CDR2L), peptidylprolyl isomerase A (PPIA), mediator complex subunit 10 (MED10), and centrosomal protein 250 (CEP250) protein expression levels detected *via* nano LC-MS/MS were confirmed in DFMO-treated cells through western blotting (Fig. 1*C*). An increased eIF5A1 expression level induced by polyamines was also observed using an anti-eIF5A1/eIF5A2 antibody, despite eIF5A1 (fold-change of None vs. DFMO: 1.531) not being identified among the 165 proteins upregulated (>2.0) by polyamines (Table S1). Given that the anti-eIF5A1/eIF5A2 antibody can simultaneously detect the two eIF5As (14, 42) and that the same Western blot analysis result was obtained using other anti-eIF5A1 antibodies, it was confirmed that the eIF5A1 expression level was also moderately upregulated by polyamines in HeLa S3 cells.

We next examined the effects of polyamine depletion on the oxygen consumption rate (OCR), an indicator of OXPHOS, and the extracellular acidification rate (ECAR), an indicator of aerobic glycolysis. Here, the reduction in the ECAR induced by DFMO treatment was greater than that of the OCR (Fig. 1, *D* and *E*). These results strongly suggest that genes modulated by polyamines activate glycolysis, rather than mitochondrial respiration, in HeLa S3 cells.

To examine the effect of polyamines on the expression levels of total RNAs, a transcriptome analysis was performed using non-treated cells (None) and DFMO-treated cells (DFMO) (Table S2). Moreover, the resulting fold-change in the expression of 16,567 RNAs in the None vs. DFMO comparison was analyzed using a volcano plot. Unexpectedly, the expression levels of only 492 (2.97%) of the RNAs were altered by polyamines (Fig. 1*F*). Among the 13,011 mRNAs (Fig. S5), the gene IDs of 5553 mRNAs and proteins were matched, and their fold-changes were analyzed using volcano plots, suggesting that the expression of proteins, rather than mRNAs, was altered by polyamines (Fig. 1, *G* and *H*). Considering the lack of a correlation between the fold-changes of proteins and mRNAs (Fig. 1*I*), these results support the idea that polyamines modulate gene expression at the translational level, stimulating cell growth (30). The proteins (5553 genes) in the None vs. DFMO comparison were classified based on the same gene ID mRNA ratio, suggesting that the expression of 238 genes, including eIF5A2, was modulated by polyamines at the translational level (Fig. 1*J*). We also examined *eIF5A1* and *eIF5A2* mRNA levels following DFMO treatment. The levels of both mRNAs were not affected by polyamine depletion (Fig. S6), confirming that the increased levels of eIF5A1 and eIF5A2 proteins induced by polyamines are due to translational regulation.

### Silencing eIF5A2, rather than eIF5A1, strongly inhibits cancer cell growth

**Figure 2:**
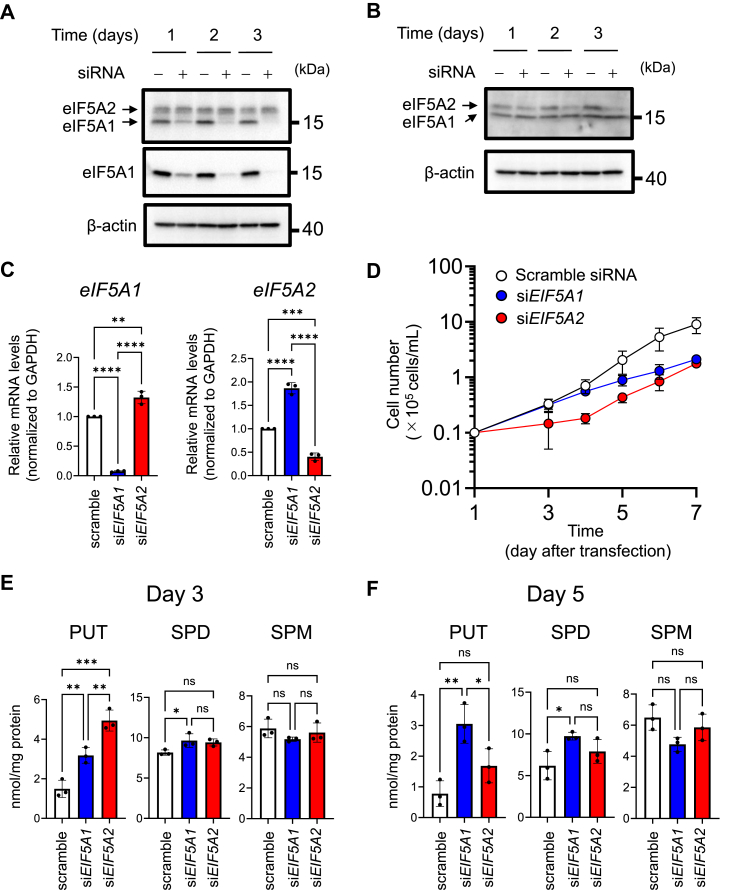
**eIF5A2 plays major roles in cell proliferation stimulated by polyamines.***A* and *B*, Western blot analysis of the two eIF5As in HeLa S3 cells treated with siRNAs against eIF5A1 (*A*) and eIF5A2 (*B*). After 24 h of transfection with siRNA, transfected cells (1.0 × 10^4^ cells/ml) were inoculated again and cultured (*n* = 3). *C*, expression levels of two *eIF5A* mRNAs in HeLa S3 cells treated with siRNA (*n* = 3). *D*, effect of silencing the expression of two eIF5As on HeLa S3 cell proliferation (*n* = 3). *E* and *F*, effect of eIF5A silencing on intracellular polyamine levels on Day 3 (*E*) and Day 5 (*F*) after transfection (*n* = 3). ∗*p* < 0.05; ∗∗*p* < 0.01; ∗∗∗*p* < 0.001; ∗∗∗∗*p* < 0.0001; ns, not significant. Experiments were repeated three times, and reproducible results were obtained.

Since polyamines influenced the expression levels of the two eIF5As (Fig. 1*C*), the effect of eIF5As on the growth of HeLa S3 cells was examined. Silencing eIF5As in HeLa S3 cells was successfully accomplished 3 days after transfection with siRNA (43, 44) (Fig. 2, *A*–*C*). Growth inhibition mediated by eIF5A2 silencing occurred 3 days after transfection, whereas decreased cell growth mediated by eIF5A1 silencing was not observed until 5 days (Fig. 2*D*). Growth inhibition induced by eIF5A2 silencing occurred after 72 h of culture, and the result was consistent with that obtained when examining cell growth following DFMO treatment (Fig. S1*A*). Moreover, no decrease in intracellular polyamine levels in cells with eIF5As knocked down was observed at 3 and 5 days after transfection (Fig. 2, *E* and *F*). Based on these findings, it is conceivable that eIF5As, particularly eIF5A2, regulated by polyamines, play major roles in the proliferation of HeLa S3 cells.

**Figure 3:**
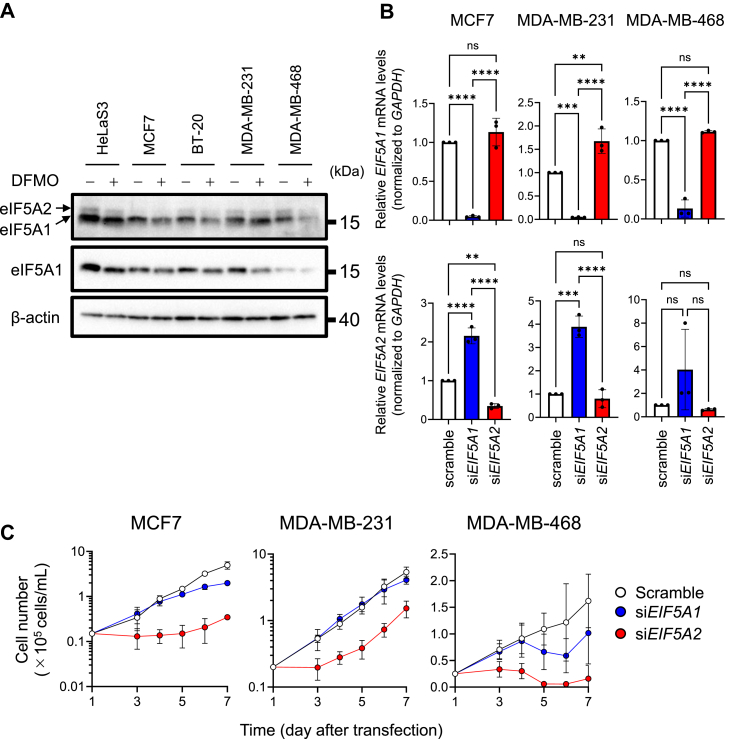
**eIF5A2 plays a key role in the proliferation of breast cancer cell lines stimulated by polyamine**. *A*, Western blot analysis of the two eIF5As in five cancer cell lines treated with or without 5 mM α-difluoromethylornithine (DFMO) (*n* = 3). 20 micrograms of cell lysate protein was used. *B*, quantitative PCR of the two *eIF5As* in MCF-7, MDA-MB-231, and MDA-MB-468 cells transfected with siRNAs. *C*, effect of eIF5A1 or eIF5A2 on the proliferation of MCF-7, MDA-MB-231, and MDA-MB-468 cells (*n* = 3). After 24 h of transfection with siRNA, transfected HeLa S3 cells were inoculated again and further cultured (*n* = 3). ∗∗*p* < 0.01; ∗∗∗*p* < 0.001; ∗∗∗∗*p* < 0.0001; ns, not significant. Experiments were repeated three times, and reproducible results were obtained.

The Kaplan–Meier curve suggested that the expression of eIF5A2, rather than eIF5A1, affects the survival of patients with breast cancer categorized by molecular subtypes (Fig. S7). DFMO treatment decreased the levels of the two eIF5As in three breast cancer cell lines, whereas the expression level of eIF5A2 in MCF7 cells was very low, and a DMFO-induced reduction was not observed (Fig. 3*A*). Although eIF5A2 silencing increased *eIF5A1* mRNA expression in breast cancer cells, including MCF7 cells (Fig. 3*B*), the inhibition of cell growth, in all three cell lines, induced by eIF5A2 silencing was stronger than that induced by eIF5A1 silencing (Fig. 3*C*). This result strongly suggests that eIF5A2, but not eIF5A1, is important for the growth of breast cancer cell lines, despite the differences in eIF5A2 expression levels. We further investigated the eIF5A2 protein expression level in other cell lines cultured in the presence or absence of DFMO. Here, eIF5A2 expression was higher in rodent cell lines (NIH3T3 and CHO-K1) than in human cell lines, whereas DFMO failed to decrease its expression in rodent cells (Fig. S8). This result suggests that the hypusination of eIF5As alone is regulated by polyamines in rodent cells.

### eIF5A2, rather than eIF5A1, affects OXPHOS and aerobic glycolysis

**Figure 4:**
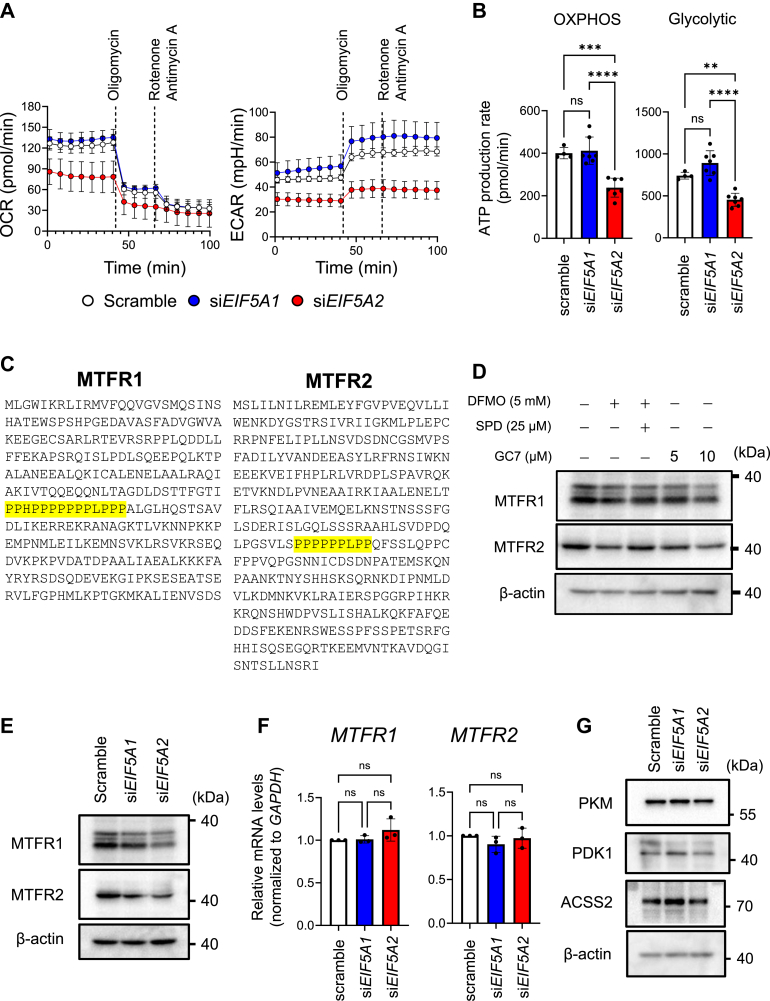
**eIF5A2, rather than eIF5A1, affects oxidative phosphorylation (OXPHOS) and aerobic glycolysis**. *A*, effect of eIF5A silencing on the oxygen consumption rate (OCR) and extracellular acidification rate (ECAR) in HeLa S3 cells (*n* = 6). The OCR and ECAR of 4.0 × 10^4^ cells/well were measured after culturing cells (1.5 × 10^4^ cells/ml) transfected with siRNA for 48 h. *B*, the ATP production rate was calculated using the OCR and ECAR measured in (*A*). ∗∗*p* < 0.01; ∗∗∗*p* < 0.001; ∗∗∗∗*p* < 0.0001. *C*, amino acid sequences of human MTFR1 and MTFR2. Polyproline motifs are shown in *yellow*. *D*, effect of α-difluoromethylornithine (DFMO) or *N*^1^-guanyl-1,7-diaminoheptane (GC7) on the expression levels of MTFR1 and MTFR2. To inhibit the hypusination of eIF5As, HeLa S3 cells were treated with 25 μM GC7 for 72 h. 20 micrograms of cell lysate protein was used. *E* and *F*, Effect of eIF5A silencing on the expression levels of MTFR1 and MTFR2 proteins (*E*) and mRNAs (*F*). It was suggested that the synthesis of MTFR1 and MTFR2 proteins was stimulated by eIF5As during translation elongation. *G*, Effect of eIF5A silencing on the expression levels of PKM, PDK1, and ACSS2 proteins. After 24 h of transfection with siRNA, transfected cells (1.0 × 10^4^ cells/ml) were inoculated again and cultured for 72 h (*n* = 3). 20 micrograms of cell lysate protein was used. *D–G*,experiments were repeated three times, and reproducible results were obtained.

We next examined the effect of eIF5A silencing on the OCR and ECAR in HeLa S3 cells cultured for 2 days after siRNA transfection. Silencing eIF5A2, but not eIF5A1, clearly reduced OCR and ECAR in HeLa S3 cells (Fig. 4, *A* and *B*). Mitochondrial fission regulator 1 (MTFR1) and MTFR2 play important roles in mitochondrial dynamics and cellular respiration (45, 46). As both have long polyproline motifs in their AA sequences (Fig. 4*C*), we investigated the effects of polyamines/eIF5As on their expression levels. Levels of MTFR1 and MTFR2 proteins were decreased following DFMO, GC7, and eIF5a silencing (Fig. 4, *D*–*F*). Moreover, the decrease in MTFR levels induced by eIF5A2 silencing was greater than that induced by eIF5A1 silencing (Fig. 4*E*); however, cell proliferation was not affected by *MTFR1* or *MTFR2* silencing (Fig. S9). These results suggest that mitochondrial dynamics related to MTFRs may not contribute to HeLa S3 cell proliferation. Next, we examined the effects of eIF5As on the expression levels of PKM, PDK1, and ACSS2 proteins, which were upregulated by polyamines. eIF5A2 silencing failed to decrease PKM, PDK1, and ACSS2 expression, whereas eIF5A1 silencing either had no effect or increased their expression (Fig. 4*G*). Thus, it remains to be clarified why eIF5A2, but not eIF5A1, activates OXPHOS and glycolysis.

### Genes upregulated by eIF5A2 are distinct from those upregulated by eIF5A1

**Figure 5:**
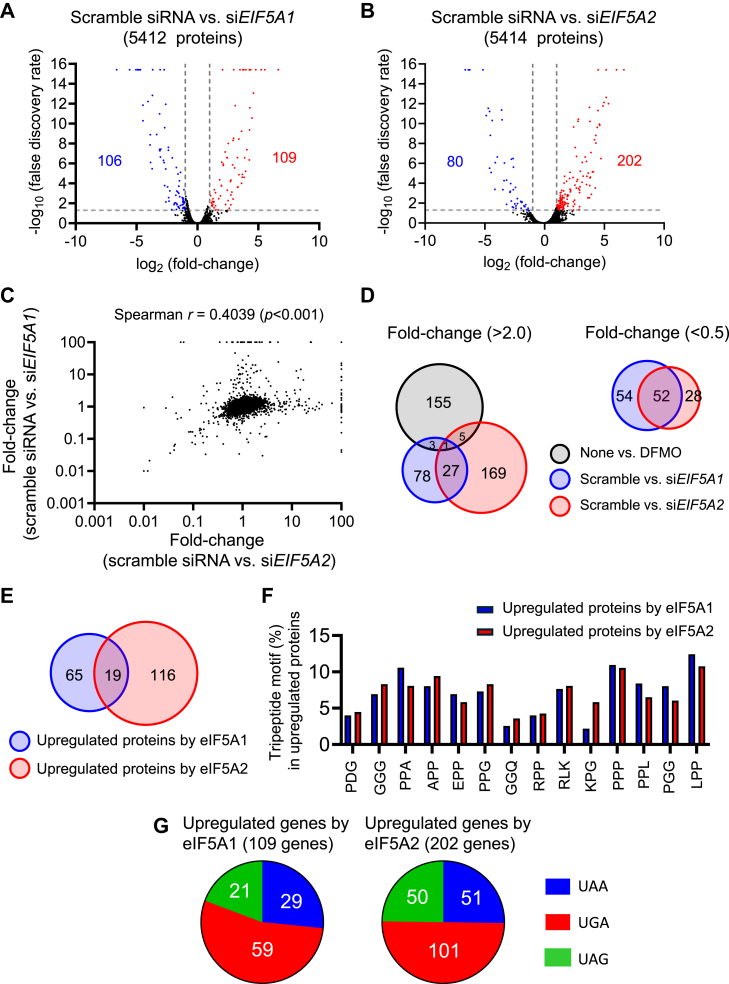
**Identification of proteins of which expression levels are regulated by eIF5As**. *A* and *B*, Volcano plot of 5412 proteins in scramble siRNA vs. si*EIF5A1* groups (*A*) and of 5414 proteins in scramble vs. si*EIF5A2* groups (*B*) (*n* = 3). *C*, uncorrelated fold-changes in individual proteins between scramble siRNA vs. siEIF5A1 and scramble siRNA *versus*. siEIF5A2 groups. *D*, Venn diagram of proteins altered (>2.0 or <0.5) by polyamines and eIF5As. *E*, Venn diagram of proteins with tripeptide motif-inducing ribosome stalling among proteins with expression upregulated by eIF5As. *F*, The number of proteins with a tripeptide motif upregulated (>2.0) by eIF5As. Tripeptide-inducing ribosome stalling was referred to as previously described by Pelechano *et al.* (8). *G*, termination codon usage in proteins with expression upregulated by eIF5As.

We next performed a proteomic analysis of HeLa S3 cells after silencing either eIF5A1 or eIF5A2 expression. The altered protein expression ratios after silencing eIF5A1 or eIF5A2 are shown in Table S3. A GO analysis of the 500 proteins most upregulated by eIF5A2 resembled those proteins upregulated by eIF5A1; in particular, levels of proteins associated with the mitochondrial membrane and rRNA transcription were upregulated (Fig. S10, *A* and *C*). However, expression levels of genes related to OXPHOS and the TCA cycle were upregulated by eIF5A2 rather than eIF5A1 (Figs. S2 and S4). A volcano plot of the geometric means of the fold-changes and the combined Stouffer’s *p*-values of 5412 proteins in the scramble *versus* si*EIF5A1* comparison and 5414 proteins in the scramble *versus* si*EIF5A2* (except for unnamed proteins) comparison showed 215 (3.99%) and 283 (5.23%) proteins (excluding eIF5A1 and eIF5A2) of which expression levels were altered by silencing eIF5A1 and eIF5A2, respectively (Fig. 5, *A* and *B*). However, no correlation was observed between the individual proteins upregulated by eIF5A2 and eIF5A1 (Fig. 5*C*). The degree of overlap of upregulated (>2.0) proteins between eIF5A1 and eIF5A2 was smaller than that of those downregulated (<0.5) by eIF5As (Fig. 5*D*). eIF5A1 participates in mRNA decoding during the elongation and termination steps and thereby prevents ribosome stalling (7, 8). Therefore, 14 tripeptide motifs (see Fig. 5*F*) that induce ribosome stalling (8) among proteins upregulated (>2.0) by eIF5As were further investigated. Although 84 (77%) of the 109 proteins upregulated by eIF5A1 and 135 (67%) of the 202 proteins upregulated by eIF5A2 had these tripeptide motifs, the Venn diagram showed that the number of proteins with tripeptide motifs upregulated by both eIF5A1 and eIF5A2 was small (Fig. 5*E*). The number of each tripeptide motif in proteins upregulated by eIF5As was further determined. The usage of the tripeptide motif (%) identified in proteins upregulated by eIF5A1 and eIF5A2 was different; particularly, the ratio of the KPG motif in proteins upregulated by eIF5A2 was higher than that in proteins upregulated by eIF5A1 (Fig. 5*F*). The function of eIF5A1 during the termination step in yeast *in vitro* translation systems was also investigated using mRNA containing the UAA (7) and UAG (8) termination codons. Therefore, the ratio of termination codons in proteins upregulated by eIF5As was also investigated, suggesting that the termination reaction with UGA and UAG might be influenced by eIF5As (Fig. 5*G*). These results suggest that eIF5A1 and eIF5A2 play distinct roles in translational elongation and termination during cell proliferation.

### Polyamines and eIF5As modulate ribosomal protein expression

**Figure 6:**
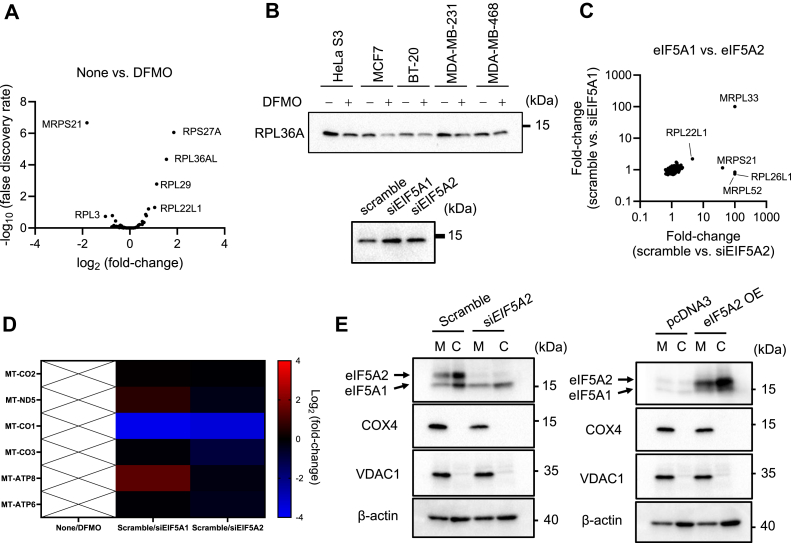
**Polyamines and eIF5As affect ribosomal components in the cytosol and mitochondria**. *A*, Volcano plot of 147 ribosomal proteins (71 cytosolic ribosomal proteins and 76 mitochondrial ribosomal proteins) obtained *via* proteomic analysis of the None *versus* α-difluoromethylornithine (DFMO) group (Table S1). *B*, effect of DFMO and eIF5A silencing on the expression level of RPL36A. 20 micrograms of cell lysate was used. *C*, Scatter plots of 147 ribosomal proteins based on the scramble siRNA vs. si*EIF5A1* and scramble siRNA vs. si*EIF5A2* comparison, obtained *via* proteomic analysis (Table S3). *D*, altered mitochondrial proteins encoded by human mtDNA affected by eIF5As silencing. Mitochondrial proteins in the None vs. DFMO groups could not be detected *via* proteomic analysis. *E*, localization of eIF5As in the mitochondrial fraction derived from HeLa S3 cells. C, cytosolic fraction; M, mitochondrial fraction. 20 micrograms of cell lysate was used. *B* and *E*, Experiments were repeated three times, and reproducible results were obtained.

Holm *et al.* reported that eIF5A1 is associated with RPL36A (eL42) and RPL10A (uL1) at the E-site of the ribosome (6). Since RPL36A is overexpressed in hepatocellular carcinoma (32) and confers radioresistance to oral squamous cell carcinoma (33), we determined whether the expression of ribosomal genes is altered by polyamines using the results of the proteomic analysis (Table S1). The expression levels of 5 proteins (7.0%), including RPL36AL, among 71 cytosolic ribosomal proteins, and MRPS21, among 76 mitochondrial ribosomal proteins, were altered by polyamines (Fig. 6*A*). Among them, RPS27A (31), RPL29 (47), and RPL22L1 (35) play a role in cancer malignancy. *RPL36AL* and *RPL36A* encode nearly identical proteins encoded by distinct genes. Thus, we confirmed that the expression level of the RPL36A protein was also decreased in polyamine-depleted HeLa S3 cells and breast cancer cell lines (Fig. 6*B*). These results raised the possibility that compositional changes in ribosomal proteins induced by polyamines facilitate interactions between eIF5A2 and cytosolic ribosomes. As RPL36A synthesis was not regulated by eIF5As (Fig. 6*B*), we next investigated the regulation of ribosomal protein expression by eIF5As using the fold-changes obtained *via* proteomic analysis (Table S3). eIF5A silencing altered the expression levels of mitochondrial ribosomal proteins rather than cytosolic ribosomal proteins (Fig. 6*C*). Moreover, eIF5As were at least partially localized to the mitochondria, and the expression level of the MT-CO1 protein, among six proteins encoded by human mtDNA, was strongly decreased by eIF5As (Fig. 6, *D* and *E*). Considering that *MT-CO1* mRNA lacks a stop codon and that mitochondrial release factor in rescue (MTRFR) is required during its translation termination (48), eIF5As might affect MTRFR-dependent translation termination in mitochondria.

### Polyamines stimulate eIF5A2 synthesis by suppressing miR-6514-5p functions

**Figure 7:**
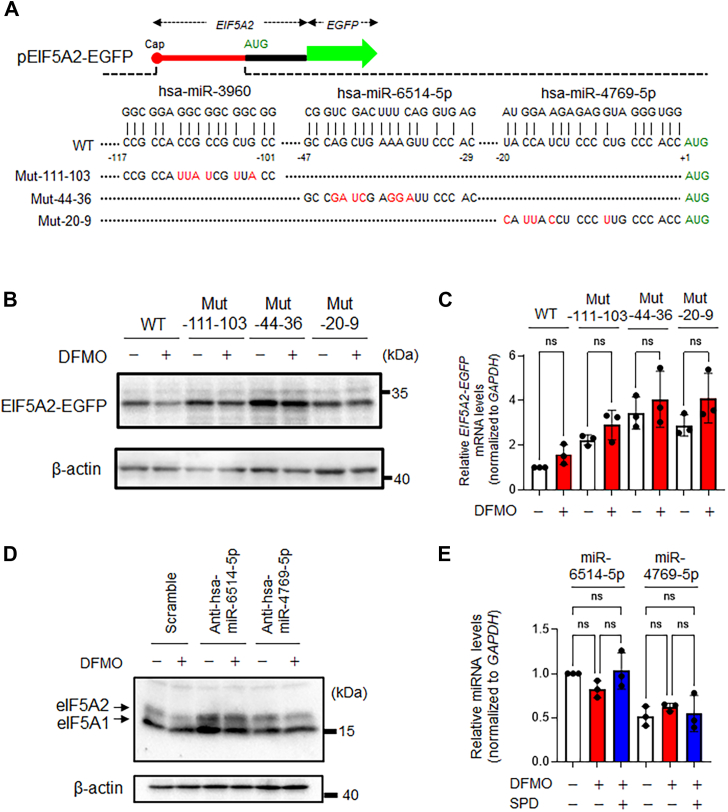
**Polyamines stimulate eIF5A2 synthesis by inhibiting hsa-miR-6514-5p function at the 5′-UTR**. *A*, possible binding sites of miRNAs at the 5′-UTR of *eIF5A2* mRNA and the structures of their mutants. The binding sites of the miRNAs were predicted using miRbase. *B* and *C*, The effect of a 5′-UTR mutation on polyamine-induced stimulation of eIF5A2-EGFP synthesis was examined *via* Western blot analysis (*n* = 3) (*B*) and qPCR (*C*). Notably, the stimulation of eIF5A2 synthesis by polyamines occurred during translation initiation, and the effect of polyamines disappeared when MUT-44 to 36 in the eIF5A2-EGFP fusion gene was used for transfection. *D*, effect of anti-miRNA on the expression level of eIF5A2 (*n* = 3). Note that when anti-hsa-miR-6514-5p was transfected into HeLaS3 cells, the expression level of the eIF5A2 protein increased and the effects of polyamine stimulation were alleviated. *E*, effects of polyamines on miRNA expression levels in HeLa S3 cells. ns, not significant. *B*–*E*, experiments were repeated three times, and reproducible results were obtained.

To elucidate the mechanism through which polyamines stimulate eIF5A2 synthesis, we prepared a reporter construct in which *eIF5A2* cDNA containing the 5ʹ UTR (130 bp) and N-terminal coding sequence (131 bp) was fused to the *EGFP* reporter gene. When HeLa S3 cells transfected with pEIF5A2 (WT)-EGFP were cultured with DFMO, decreased levels of the eIF5A2-EGFP fusion protein, but not its mRNA, were observed (Fig. 7, *A*–*C*). These results suggest that the stimulation of eIF5A2 synthesis mediated by polyamines occurs at the translation initiation step. It has been previously reported that the translation initiation of exostosin 2 (*EXT2*) mRNA is negatively regulated by miRNA let-7b and that polyamines inhibit let-7b-mediated gene suppression, facilitating EXT2 synthesis (49). A miRBase (https://www.mirbase.org/) search identified three potential miRNA-binding sites in the 5ʹ UTR of *eIF5A2* mRNA (Fig. 7*A*). Subsequently, we constructed three mutants that cannot interact with miRNAs (Fig. 7*A*). Among these mutants, the expression level of the eIF5A2-EGFP fusion protein produced from the eIF5A2 mutant (−44–36), which harbors mutations in the miR6514-5p-binding site, was increased, and the sensitivity of this mutant to DFMO was reduced compared to that of the WT mRNA (Fig. 7*B*). Since the expression level of Mut (−44–36) mRNA was also higher than that of WT mRNA (Fig. 7*C*), the effect of anti-hsa-miR6514-5p on the polyamine-induced stimulation of intrinsic eIF5A2 synthesis was examined. As a result, the expression level of eIF5A2 was increased, and DFMO sensitivity was reduced (Fig. 7*D*). The expression level of miR6514-5p was not affected by DFMO treatment (Fig. 7*E*). These results indicate that the translation initiation of *eIF5A2* mRNA is negatively regulated by miR-6514-5p and that polyamines release this miRNA suppression, facilitating eIF5A2 synthesis.

## Discussion

Intracellular polyamine levels are strictly regulated through biosynthesis, degradation, and transport mechanisms. Changes in polyamine metabolism are correlated with various pathologies, including cancer and age-related conditions. The activation of mitochondrial respiration by polyamines and eIF5A1 *via* upregulation of the expression of autophagy-related genes results in anti-senescence effects in aged cells (17, 18, 22, 23). However, how polyamines directly modulate genes that affect cancer progression, in particular aerobic glycolysis-dependent cell growth, remains unclear. Here, findings of a proteomic analysis suggested that gene expression modulated by polyamines results in the activation of glycolysis, rather than mitochondrial respiration. In particular, expression levels of PDK1 and PKM, which are essential for glycolysis-dependent cell growth (37, 50), were upregulated by polyamines. In addition, eIF5A2 synthesis was also stimulated by polyamines at the translational level. Notably, eIF5A2, but not eIF5A1, was found to play a major role in human cancer cell proliferation. Our proteomic analysis suggested that individual proteins upregulated by eIF5A2 were distinct from those upregulated by eIF5A1. These results strongly suggest that eIF5A1 and eIF5A2 play distinct roles during cell proliferation.

However, it remains unclear why despite being 84% identical, eIF5A2 participates in mRNA decoding in a distinct manner compared to eIF5A1. Moreover, they both have similar hypusination efficiencies mediated by DHS and protein stability (3, 4, 51). Therefore, we focused on the structural differences between eIF5A1 and eIF5A2. Holm *et al.* reported that eIF5A1 occupies the E-site and stabilizes P-site tRNA for efficient peptide bond formation (6). Hypusinated Lys^50^ supports 3ʹ-CCA interactions with the peptidyl transferase center in the initiation complex (IC) of ribosomes during the elongation step (6). In addition, the C-terminus of eIF5A1 interacts with the RPL10A of the large subunit (LSU) of the ribosome, and the N-terminus of eIF5A1 wedges against the P-site tRNA D loop, RPL36A, and LSU rRNA helix74 in the IC state (6). The AA sequences of eIF5A1 and eIF5A2 differ by 28 residues, of which, 22 are located in the C-terminus after Gln^90^. Since the C-terminus of eIF5A1 associates with RPL10A *via* hydrogen bonding, hydrophobic interactions, or electrostatic interactions (Table S4), molecular dynamic (MD) simulations were conducted to determine the three-dimensional structure of eIF5A1–RPL10A and eIF5A2–RPL10A complexes. The MD analysis revealed 100 structures, suggesting that 35 AA residues of the two eIF5As associated with RPL10A; however, 17 of these in eIF5A1 bound to RPL10A more frequently than those in eIF5A2 (Fig. S11*A*). A comparison of the secondary structures of the two eIF5As suggested that eIF5A2 had an increased frequency of beta-sheet structures at Asp^95^, Val^108^, and Arg^109^. Moreover, frequency of the alpha-helix formed by Gly^117^–Lys^126^ in eIF5A2 was higher than that in eIF5A1 (Table S5). These changes were observed in the parallel beta-sheet structure formed by Lys^85^–Arg^113^ and the alpha-helix structure formed by Gly^117^–Asp^128^, which were folded in eIF5A2. Furthermore, these residues in eIF5A1 exhibited an increased frequency of loop and turn structures, suggesting that the secondary structures of the C-terminus of eIF5A2 were more likely to be folded than those of eIF5A1, resulting in reduced binding to RPL10A (Figs. S11 and S12). These results support the idea that polyamine-mediated alterations in ribosome components facilitate more frequent associations between ribosomes and eIF5A2 than with eIF5A1 during cancer progression (Fig. S13).

Our results demonstrated the major role of eIF5A2 in the polyamine-mediated stimulation of cell growth; however, no correlations between fold-changes in the None vs. DFMO and those in the scramble vs. siEIF5As comparison (Fig. S8, *E* and *F*) were found, implying that genes modulated by polyamines are also distinct from those regulated by eIF5As. There are several reports indicating that the synthesis of antizymes (52, 53), eEF1A (54), EXT2 (49), chondroitin synthase 1 (CHSY1) (55), histone acetyltransferases (56), and circadian clock proteins (57) is enhanced by polyamines at the translational level. Notably, the characteristic RNA structures in their mRNA result in inefficient translation, and polyamines facilitate translation initiation *via* structural changes in mRNAs (49, 52, 53, 54, 55, 56, 57). Given that the expression level of PKM was not downregulated significantly after eIF5As silencing (Fig. 4*G*), their expression might be regulated by polyamines through the induction of structural changes in their mRNA. Based on these observations, we propose that polyamine-led glycolysis activation during cancer progression is based on the combination of altered gene expression *via* structural changes in specific mRNAs and the increased eIF5A2 translation elongation, both regulated by polyamines.

Notably, we found that RPL22L1 synthesis was upregulated by polyamines, as well as eIF5A1 and eIF5A2 (Fig. 6, *A* and *C*). Although RPL22L1 expression is induced in a compensatory manner in Rpl22^−/−^ mice and can support translation (58), RPL22L1 plays a pivotal role in the malignancy of colorectal cancer (34), hepatocellular carcinoma (35), and prostate cancer (36). However, the role of RPL22L1 during mRNA decoding with eIF5A2 in cancer cells remains unclear. Experiments are in progress to clarify the polyamine-stimulatory mechanisms of eIF5A1, PKM, RPL36A, and RPL22L1 synthesis and functions of RPL36A and RPL22L1 during translation elongation in cancer cells. It should be noted that translational fidelity regulated by polyamines (59) may influence the absence of correlations between fold-changes in None vs. DFMO and those in the scramble vs. siEIF5As comparisons. Polyamine depletion may trigger the incorporation of the wrong AA or a shift of the reading frames, resulting in the upregulation of ER stress (40, 41).

The upregulation of eIF5A2 expression mediated by hypoxia (60), reactive oxygen species-related pathways (61), and TGFβ signaling (62) exacerbates cancer prognosis. However, Clement *et al.* detected eIF5A1 protein expression in all human cancer cell lines, whereas eIF5A2 expression was cell type-specific, despite *eIF5A2* mRNA being constitutively expressed (51). In this report, *eIF5A2* mRNA was found to contain four polyadenylation signals used to generate multiple mRNAs (0.7–5.6 kb) that encode identical proteins, and cis-elements in the 5- or 3′-UTR may trigger inefficient translation (51). The results are supported by the fact that eIF5A2 expression is negatively regulated by miR203 (63) and miR577 (64) *via* an interaction with the 3′-UTR, and the loss of miRNA function is involved in the progression of human tumors. Our results suggest that the translation initiation of *eIF5A2* mRNA is suppressed by hsa-miR6514-5p *via* an interaction with the 5′-UTR and that polyamines inhibit miRNA functions, stimulating eIF5A2 synthesis in HeLa S3 cells. As previously mentioned, the translation initiation of *EXT2* mRNA is negatively regulated by the miRNA let-7b, and polyamines inhibit let-7b-mediated gene suppression to promote EXT2 synthesis (49). The addition of SPD decreased the level of *EXT2* mRNA in the anti-AGO2 antibody immunoprecipitate, suggesting that it hindered the binding of miRISC to *EXT2* mRNA (49). We speculate that the structural change in the eIF5A2 5′-UTR mediated by polyamines affects the polyamine-mediated stimulation of eIF5A2 synthesis because the amount of polyamine bound is 2 to 6.5 mol/100 mol of RNA phosphate in cells (28, 29). Further studies are needed to understand the physiological function of miR6514-5p as a tumor suppressor gene and to evaluate its usefulness as a new potential therapeutic target for tumor progression.

To conclude, gene expression modulated by polyamines activates glycolysis in HeLa S3 cells. In addition, silencing eIF5A2, rather than eIF5A1, strongly inhibits cancer cell growth, and genes upregulated by eIF5A2 are distinct from those upregulated by eIF5A1. These results strongly suggest that eIF5A2 potentially participates in the ribosomal machinery involved in cancer progression and that eIF5A2 and its binding site in the ribosome are selective targets for cancer treatment.

## Experimental procedures

### Materials

Putrescine dihydrochloride, spermidine trihydrochloride, and spermine tetrahydrochloride were obtained from Nacalai Tesque Inc. DFMO was obtained from Marion Merrell Dow Research Institute. GC7 was kindly supplied by Dr M. H. Park, National Institutes of Health. All other chemicals were of analytical grade.

### Cell culture

HeLa S3, BT-20, MDA-MB-231, MDA-MB-468, MCF7, HEK293, NIH3T3, and CHO-K1 cells were purchased from the American Type Culture Collection. All cells, except BT-20, were cultured in Dulbecco’s Modified Eagle Medium (DMEM; Nacalai Tesque, Inc) supplemented with 10% fetal bovine serum (FBS; Thermo Fisher Scientific Inc), 100 units/ml penicillin G, and 50 units/ml streptomycin (Nacalai Tesque, Inc) in an atmosphere of 5% CO_2_/95% air at 37 °C. BT-20 cells were cultured in Eagle’s minimum essential medium (Fujifilm Wako Pure Chemical Corporation) supplemented with 10% FBS. A three-fold higher number of cells was cultured in DMEM in the presence of 5 mM DFMO for 3 days to produce polyamine-depleted cells. When 25 μM of SPD was added to DFMO-treated cells in a culture medium containing 10% FBS, 1 mM aminoguanidine, an inhibitor of Cu^2+^-dependent amine oxidase (65), was also added to protect against cellular damage mediated by H_2_O_2_ and acrolein generated during SPD catabolism.

### Determination of polyamine contents

Cells (1.0 × 10^6^ cells) were suspended in 100 μl of 5% trifluoroacetic acid and incubated at 70 °C for 10 min. After centrifugation at 15,000*g* for 10 min at 4 °C, the resulting supernatant was subjected to high-performance liquid chromatography (HPLC). The pellet obtained following centrifugation was dissolved in 1 M NaOH and used to measure the protein content using the method described by Lowry *et al.* (66). Polyamines were determined using HPLC with fluorescence detection, according to the methods described by Miyajima *et al.* (41).

### Proteome analysis

HeLa S3 cells (3.0 × 10^6^) treated with or without DFMO or siRNA were dissolved in 7 M guanidine-HCl, 1 M Tris-HCl (pH 8.5), 10 mM EDTA, and 50 mM dithiothreitol. After alkylation with 100 mM acrylamide, the samples were desalted using a PAGE Clean Up Kit (Nacalai Tesque, Inc). The resultant precipitates were dissolved in 20 mM Tris-HCl (pH 8.0), 0.03% (w/v) n-dodecyl-β-_D_-maltoside and digested with trypsin (tosyl phenylalanyl chloromethyl ketone-treated; Worthington Biochemical Co) at 37 °C overnight. Three technical replicates of each peptide were subjected to LC-MS/MS using an Easy nLC 1200 (Thermo Fisher Scientific Inc) and Q Exactive HF-X Mass Spectrometer (Thermo Fisher Scientific Inc.). Solvents A (0.1% formic acid) and B (80% acetonitrile with 0.1% formic acid) were used as the LC solvents. Peptides were separated using a nano-ESI spray column (NTCC-360, 0.075 mm internal diameter × 150 mm length, 3 μm, Nikkyo Technos Co) at a flow rate of 300 nl/min under a linear gradient condition from 0% B to 40% B in 220 min. The mass spectrometer was operated in the positive mode using the data-dependent Top 10 method. The acquired data were processed using MASCOT 2.8 (Matrix Science, London, U.K.) and Proteome Discover 3.0 (Thermo Fisher Scientific Inc). The MASCOT search was conducted as follows: database, NCBIprot; taxonomy, *Homo sapiens* (human); type of search, MS/MS ion; enzyme, trypsin; fixed modification, propionamide (C); variable modifications, acetyl (protein N-term), Gln->pyro-Glu (N-term Q), oxidation (M); mass values, monoisotopic; peptide mass tolerance, ±15 ppm; fragment mass tolerance, ±30 mmu; max missed cleavages, 3; instrument type, ESI-TRAP. Label-free quantification was performed using the Proteome Discoverer.

### Western blot analysis

Western blotting was performed as described by Nielsen *et al.* (67) using Chemi-Lumi One Ultra (Nacalai Tesque). The antibodies used in this study are listed in Table S6. Each antibody was diluted with Signal Enhancer HIKARI (Nacalai Tesque) or 1× TBS-T buffer (10 mM Tris-HCl, 150 mM NaCl, 0.05% Tween20). Protein levels were quantified using the ChemiDoc MP Imaging System (Bio-Rad Laboratories, Inc). The protein content was determined using the method described by Lowry *et al.* (66).

### Measurement of mRNA

Total RNA was isolated using the RNeasy Mini Kit (Qiagen GmbH) according to the manufacturer’s instructions. cDNA was synthesized from 1 μg of RNA using Prime Script RT Master Mix (TaKaRa Bio Inc). RNA sequencing was performed at MacroGen Inc (www.macrogen.com) using a NovaSeq 6000 system. Library construction and sequencing were performed according to standard sequencing protocols recommended by Illumina. All primers used for mRNA measurements are listed in Table S7. Quantitative PCR (qPCR) with TB Green Premix Ex Taq II (TaKaRa Bio Inc) was performed using primers P1 and P2 to measure the transcript levels of *EIF5A1*. The primers used to amplify *EIF5A2* were P3 and P4. Primers used to amplify *MTFR1* were P5 and P6. The primers used to amplify *MTFR2* were P7 and P8. Transcript levels were calculated using the 2^−ΔΔCt^ method. Transcription of the housekeeping gene *GAPDH* was used to normalize the data. The primers used to amplify *GAPDH* were P9 and P10.

### Plasmid construction

All primers used for the construction of pEIF5A2-EGFP, pEIF5A2-EGFP mutants, pcDNA-EIF5A1, and pcDNA-EIF5A2 are listed in Table S7. PCR was performed using the following primer set, P#1 and P#2, P#3, and P#4, P5, and P6. Furthermore, cDNA, prepared as described previously, was used as a template to amplify *EIF5A2* containing the 5′-UTR and N-terminal coding sequence (CDS) (261 bp), CDS of *EIF5A1* (479 bp), and CDS of *EIF5A2* (476 bp). The amplified *EIF5A2* gene comprising 5′-UTR and N-terminal CDS was digested with EcoRⅠ (TOYOBO Co, Ltd) and BamHⅠ (TOYOBO CO, LTD). The digest was inserted into the same restriction site of pEGFP-N1 (TaKaRa Bio Inc). The amplified CDSs of *EIF5A1* and *EIF5A2* corresponding to the open reading frames were digested with HindIII (TOYOBO Co., Ltd) and XbaI (New England Biolabs Japan Inc.) and inserted into the same restriction site of pcDNA3 (Thermo Fisher Scientific Inc). Overlap extension PCR was performed using the primers listed in Table S7, and pEIF5A2-EGFP was used as a template to generate pEIF5A2-EGFP mutants [pEIF5A2 (Mut-111-103)-EGFP, pEIF5A2 (Mut-44-36)-EGFP, and pEIF5A2 (Mut-20-9)]. Plasmid sequences were confirmed through DNA sequencing (Eurofins Genomics K.K.).

### Gene knockdown

Silencer select siRNAs for eIF5A1 were purchased from Thermo Fisher Scientific Inc. MISSION siRNA for eIF5A2, MTFR1, and MTFR2 were purchased from Merck Millipore Ltd. The following siRNA sequences (sense/antisense) were used in this study: 5′-GGUCCAUCUGGUUGGUAUU[dT][dT]-3′/5′-AAUACCAACCAGAUGGACC[dT][dT]-3′ for eIF5A1 (43), 5′-GGAUCUUAAACUGCCAGAA[dT][dT]-3′/5′-UUCUGGCAGUUUAAGAUCC[dT][dT]-3′ for eIF5A2 (44), 5′-GAGUUCAGUUUCAGAUUAA[dT][dT]-3′/5′-UUAAUCUGAAACUGAACUC[dT][dT]-3′ for MTFR1, and 5′-CCUGUAAAUGAAGCUGCAA[dT][dT]-3′/5′-UUGCAGCUUCAUUUACAGG[dT][dT]-3′ for MTFR2. Silencer Select Control No.1 siRNA (catalog no. 4390843) was used as a scrambled control. The transfection of siRNA was performed using Lipofectamine RNAiMAX (Thermo Fisher Scientific Inc) according to the manufacturer’s instructions. Briefly, 30 pmol of siRNA was mixed with 4 μl of Lipofectamine RNAiMAX in 500 μl of Opti-MEM and allowed to stand for 20 min at room temperature. HeLa S3 (1.5 × 10^5^ cells), MCF7 cells (2.5 × 10^5^ cells), MDA-MB-231 (1.5 × 10^5^ cells), and MDA-MB-468 (1.5 × 10^5^ cells) were inoculated into 2 ml of DMEM with 10% FBS in 6-well plates and cultured for 24 h. After replacing the medium with a fresh one, cells were transfected with 500 μl of siRNA/Lipofectamine RNAiMAX complex in Opti-MEM and cultured in DMEM with 10% FBS. After culturing for 24 h, the transfected cells were collected following trypsin treatment (0.25 w/v% Trypsin-1 mmol/L EDTA.4Na solution) (Nacalai Tesque, Inc). Transfected cells (1 × 10^4^ cells/ml) inoculated and further cultured for more than 2 days were used for the experiments.

### Transfection of plasmids and miRNA inhibitor

The transfection of plasmids into HeLa S3 cells was performed as described by Fukumoto *et al.* (68) with minor modifications. Briefly, 1 μg of plasmids was mixed with 6 μg of polyethyleneimine (Polysciences, Inc) in 100 μl of buffer (20 mM sodium lactate, 150 mM NaCl [pH 4.0]) and allowed to stand for 20 min at room temperature. Subsequently, 500 μl of Opti-MEMⅠ (Thermo Fisher Scientific Inc) was added. Next, 1.6 × 10^5^ cells in 6-well plates were cultured for 24 h. After changing the medium to a fresh one containing FBS, the cells were transfected with 600 μl of a plasmid/polyethyleneimine complex in Opti-MEM and cultured in DMEM containing FBS for 8 h. After replacing the culture medium with a fresh medium, the cells were cultured with or without 5 mM DFMO for 40 h. The transfection of an miRCURY LNATM miRNA inhibitor for hsa-miR-6514-5p (Qiagen GmbH) or hsa-miR-4769-5p (Qiagen GmbH) or siRNA Silencer select Control No.1 siRNA (Thermo Fisher Scientific Inc., Bartlesville) was performed using lipofectamine 2000 (Thermo Fisher Scientific Inc, Bartlesville) according to the manufacturer’s protocol.

### Preparation of mitochondrial fraction

Scramble siRNA and si*EIF5A2* were transfected using Lipofectamine RNAiMAX (Thermo Fisher Scientific Inc) according to the manufacturer’s instructions. Briefly, 150 pmol siRNA was mixed with 20 μl Lipofectamine RNAiMAX in 2.5 ml Opti-MEM and allowed to stand for 20 min at room temperature. HeLa S3 cells (7.5 × 10^5^ cells) were inoculated into 10 ml DMEM containing 10% FBS in 10 cm plates and cultured for 24 h. After replacing the medium with a fresh medium, cells were transfected with 2.5 ml siRNA/Lipofectamine RNAiMAX complex in Opti-MEM and cultured in DMEM with 10% FBS. After culturing for 72 h, the transfected cells were collected following trypsin treatment (0.25 w/v% Trypsin-1 mmol/L EDTA.4Na solution; Nacalai Tesque, Inc).

pcDNA3 and pcDNA/EIF5A2 were transfected into HeLa S3 cells as described by Fukumoto *et al.* (68) with minor modifications. Briefly, 10 μg of plasmids was mixed with 60 μg of polyethyleneimine (Polysciences, Inc) in 1 ml of buffer (20 mM sodium lactate, 150 mM NaCl [pH 4.0]) and allowed to stand for 20 min at room temperature. Subsequently, 5 ml of Opti-MEMⅠ (Thermo Fisher Scientific Inc) was added to the mixture. Next, 7.5 × 10^5^ cells in 10 cm plates were cultured for 24 h. After changing the medium to a fresh medium containing FBS, the cells were transfected with 6 ml of the plasmid/polyethyleneimine complex in Opti-MEM and cultured in DMEM containing FBS for 8 h. After replacing the culture medium with a fresh medium, the cells were cultured for another 64 h. The transfected cells were collected following trypsin treatment (0.25 w/v% Trypsin-1 mmol/L EDTA.4Na solution; Nacalai Tesque, Inc).

Mitochondria and cytosol fractions were prepared using a Mitochondria/Cytosol Fraction Kit (BioVision Inc) according to the manufacturer’s instructions. Briefly, 5.0 × 10^7^ cells were resuspended in 1 ml of 1 × Cytosol Extraction Buffer Mix containing a protease inhibitor cocktail and DTT. After that, these samples were incubated on ice for 10 min and homogenized with a Power Masher II (Nippi Inc.). Homogenate samples were centrifuged at 3000 rpm for 10 min at 4 °C. The resulting supernatants were transferred to a new 1.5 ml plastic tube and centrifuged at 13,000 rpm for 30 min at 4 °C. The obtained supernatant was used as the cytosolic fraction. The pellets were resuspended 100 μl of the Mitochondrial Extraction Buffer Mix containing DTT and protease inhibitors, vortexed for 10 s, and collected as the mitochondrial fraction. The expression of eIF5As, COX2, and VDAC1 in each fraction was confirmed *via* western blotting. 20 micrograms of each fraction of protein were used.

### Measurement of miRNA

Total RNA was isolated using the miRNeasy Mini Kit (Qiagen GmbH, Hilden, Germany) according to the manufacturer’s instructions, and cDNA was synthesized from 2 μg of RNA using the Mir-X miRNA First-Strand Synthesis Kit (Takara Bio Inc). qPCR with TB Green Premix Ex Taq II (TaKaRa Bio Inc) was performed using the primer P13 to measure the transcriptional level of the *miR-6514-5p* gene. The P14 primers were used to amplify the *miR-4769-5p* gene. All primers used for miRNA measurements are listed in Table S7. Transcript levels were calculated using the 2^−ΔΔCt^ method. Transcription of the housekeeping gene *GAPDH* was used to normalize the data. The primers used to amplify *GAPDH* were 5′-AGCCACATCGCTCAGACAC-3′ (P11) and 5′-GCCCAATACGACCAAATCC-3′ (P12).

### Analysis of the molecular taxonomy of breast cancer International Consortium (METABRIC) dataset

The METABRIC dataset (*n* = 2509) (69, 70) was downloaded from the cBioportal (https://www.cbioportal.org/) Analysis of the METABRIC dataset on July 21, 2022. The clinicopathological data of these patients have been previously summarized (71). The METABRIC dataset contains data on both gene changes (*n* = 2173) and mRNA expression levels in primary breast cancer samples (*n* = 1904), as well as overall survival (OS) data with *EIF5A1* mRNA expression levels (*n* = 1419) and OS data with *EIF5A2* mRNA expression levels (*n* = 1369).

### Cellular respiration analysis

The cells were dissociated using Accutase and resuspended in high-glucose DMEM. The cells were then seeded in an Agilent Seahorse XF24 cell culture microplate (Agilent Technologies; 4.0 × 10^4^ cells/well), and the plates were incubated in an atmosphere of 5% CO_2_/95% air at 37 °C for 3 h. The culture medium was replaced with an analysis medium and high-glucose XF DMEM (Cat#103575-100, Agilent Technologies). Subsequently, the OCR and ECAR were analyzed using an Agilent Seahorse XFe24 analyzer (Agilent Technologies). The analysis medium comprised 2 mM Seahorse XF glutamine solution (Agilent Technologies), 1 mM Seahorse XF pyruvate solution (Agilent Technologies), and 25 mM glucose in the high-glucose medium. OCR and ECAR were analyzed 1 h after replacement with fresh high-glucose DMEM. Additionally, the rate of ATP production was analyzed using the Seahorse XF Real-Time ATP Rate Assay Kit (Cat#103592-100, Agilent Technologies) according to the manufacturer’s protocol.

### Computational analysis

Three-dimensional structures of eIF5A1 and RPL10A were obtained from the RSCB PDB (PDBID: 8G61). The eIF5A2 structure was derived from the eIF5A1 structure using homology modeling in Discovery Studio (Dassault Systems Co., Ltd). Next, all-atom MD simulations were performed using Amber16, and the parameter sets were modeled in the ff14SB force field. Each structure was placed in a periodic box of TIP3P water with an 8 Å solvent buffer between the structure and the edge of the box. Energy minimization was performed with a constant volume of 10,000 steps, consisting of 2000 steepest descents and 8000 conjugate gradients. Heating was performed from 0 to 310 K. Equilibration was performed using the NPT ensemble for 2 ns. The cutoff distance for non-bonded pair interactions was 10 Å. Finally, a production simulation was performed for 100 ns trajectories every 1.0 ns under 310 K and 1.0 atm conditions.

### Statistical analysis

For the METABRIC analysis, mRNA expression levels were compared among breast cancer subtypes using the Kruskal–Wallis test, followed by Steel–Dwass’s multiple comparison test for the *post hoc* analysis. The optimal cutoff thresholds to divide patients into groups with high and low expression were defined using receiver operating characteristic curves based on the association between *EIF5A1* or *EIF5A2* gene expression and disease-specific survival (DSS). The optimal cutoff threshold was determined using Youden’s index. Survival curves based on OS were plotted using the Kaplan–Meier method, and they were compared using the log-rank (Cochran–Mantel–Haenszel) test. Multivariate Cox regression analysis was used to evaluate the influence of gene expression and estimate adjusted hazard ratios based on the DSS status, with age at diagnosis as a confounding factor. Two-sided values of *p* < 0.05 were considered statistically significant. Statistical analyses were performed using GraphPad Prism software version 10.4.2 (GraphPad Software).

For the remaining analyses, values are indicated as means ± SD. The significance of differences between the two groups was analyzed using Student’s *t*-tests. One-way analysis of variance followed by Dunnett’s test was used to evaluate the significant differences between the groups treated with or without DFMO/DFMO+SPD. All statistical analyses were performed using GraphPad Prism version 10.4.2 (GraphPad Software).

## Data availability

The data that support the findings of this study are available from the corresponding author upon reasonable request. The mass spectrometry proteomic data have been deposited into the ProteomeXchange Consortium *via* the PRIDE database with the dataset identifier PXD063151.

## Supporting information

This article contains supporting information.

## Conflict of interest

The authors declare that they have no conflicts of interest with the contents of this article.
