# Supplementary material for: Polyamines stimulate the protein synthesis of the translation initiation factor eIF5A2, participating in mRNA decoding, distinct from eIF5A1
Source: J Biol Chem. 2025 Jul 4;301(8):110453. doi: 10.1016/j.jbc.2025.110453 (PMC12329600; doi:10.1016/j.jbc.2025.110453)
Supplement: Supporting information [file mmc1.docx]

**Figure S1. Effect of α-difluoromethylornithine (DFMO) and DFMO+spermidine (SPD) on cell growth and intracellular polyamines in HeLa S3 cells.**

*A*, Effects of DFMO and SPD on HeLa S3 cell growth. After 12 h of cell culture (1.0 × 10^4^ cells/mL), 5 mM DFMO ± 25 μM SPD was added to the culture medium, and cells were cultured (*n* = 3). *B*, Intracellular polyamine levels in non-treated HeLa S3 cells (None), DFMO-treated cells (DFMO), and DFMO+SPD-treated cells (DFMO+SPD) (*n* = 3). Amounts of polyamines were normalized to those of cell lysate proteins.

**Figure S2. Categories of proteins significantly altered by polyamines in HeLa S3 cells.**

Heat maps showing differential changes (log_2_ fold expression) in the expression levels of proteins based on non-treated vs. α-difluoromethylornithine (DFMO) (None/DFMO), scramble siRNA vs. siEIF5A1 (scramble/siEIF5A1), and scramble siRNA vs. siEIF5A2 (scramble/siEIF5A2) comparisons; *n* = 3 samples per group. The 300, 215, and 282 proteins significantly altered by polyamines, eIF5A1, and eIF5A2 (Figs. 1*B*, 5A, and 5*B*) were categorized based on KEGG pathways, Reactome Pathways, and Wiki pathways. Proteins corresponding to glycolysis, oxidative phosphorylation (OXPHOS), autophagy, and the TCA cycle are shown.

**Figure S3. Top 10 Gene Ontology (GO) terms for categories (biological process) that are significantly enriched in the 500 most regulated proteins by polyamines in HeLa S3 cells.**

Proteins upregulated (*A*) and downregulated (*B*) by polyamines.

**Figure S4.** **Categories of proteins altered by polyamines in HeLa S3 cells.**

Heat maps showing differential changes (log_2_ fold) in the expression levels of proteins based on None vs. α-difluoromethylornithine (DFMO) (None/DFMO), scramble siRNA vs. siEIF5A1 (scramble/siEIF5A1), and scramble siRNA vs. siEIF5A2 (scramble/siEIF5A2) comparisons; *n* = 3 samples per group. The detected proteins (except for proteins shown in Fig. S2) via proteomic analysis (Tables S1 and S3) were categorized based on KEGG pathways, Reactome Pathways, and Wikipathways. Proteins corresponding to glycolysis, oxidative phosphorylation (OXPHOS), autophagy, and the TCA cycle are shown.

**Figure S5. Effect of polyamines on the expression levels of mRNA, pseudogene RNA, lncRNA, miRNA, and tRNA in HeLa S3 cells.**

Volcano plot of mRNAs (*A*), pseudogene RNA (*B*), lncRNA and miRNA (*C*), and tRNA (*D*). (*A*) Fewer than 3.5% of the genes were regulated by polyamines at the transcriptional level.

**Figure S6.** **Levels of eIF5A1 and eIF5A2 mRNA in HeLa S3 cells treated with α-difluoromethylornithine (DFMO) or DFMO+spermidine (SPD).** The effects of polyamines on the expression levels of *eIF5A1* and *eIF5A2* mRNA were examined using qPCR.

**Figure S7. High expression of eIF5A2, rather than eIF5A1, contributes to poor prognosis in breast cancer.**

*A*, Kaplan–Meier analyses of disease-specific survival (DSS) in patients with high expression of eIF5A1. *B*, Kaplan–Meier analyses of DSS in patients with high expression of eIF5A2.

**Figure S8. Levels of eIF5As in human and rodent cell lines treated with or without α-difluoromethylornithine (DFMO).**

Western blot analysis of eIF5A1 and eIF5A2 was performed using 20 μg of cell lysate proteins (*n* = 3). To reduce intracellular polyamine levels, animal cell lines were treated with 5 mM DFMO for 72 h.

**Figure S9. Effect of mitochondrial fission regulator 1 (*MTFR1*) and *MTFR2* gene silencing on the growth of HeLa S3 cells.**

Western blot analysis (*A*, *C*) and qPCR (*B*, *D*) of MTFRs in HeLa cells transfected with siRNA for eIF5As. Western blot analysis was performed using 20 μg of cell lysate proteins (*n* = 3). (*E*) Effect of gene silencing of MTFR1 and MTFR2 on proliferation of HeLa S3 cells. Data are expressed as the mean ± S.D. (*error bars*). ***p* < 0.01; *****p* < 0.0001; ns, not significant.

**Figure S10. Gene ontology (GO) analysis of proteins regulated by eIF5A1 and eIF5A2 in HeLa S3 cells.**

*A* and *B*, Top 10 GO terms for categories (biological process) significantly enriched based on the 500 most upregulated proteins (*A*) and downregulated proteins (*B*) by eIF5A1. *C* and *D*, Top 10 GO terms for categories (biological process) significantly enriched based on the 500 most upregulated proteins (*C*) and downregulated proteins (*D*) by eIF5A2. *E* and *F*, Scatter plots of fold-changes in None vs. α-difluoromethylornithine (DFMO) and scramble vs. si*EIF5A1* (*E*) and None vs. DFMO and scramble vs. si*EIF5A2* (*F*) comparisons.

**Figure S11. Analysis of the 100 structures obtained from molecular dynamic (MD) simulations of the interaction between eIF5As and RPL10A.**

Holm *et al.* obtained the three-dimensional structure of each state, initiation state (IC: 8G5Y), codon recognition state (CR: 8G60), GTPase-activated state (GA: 8G5Z), and accommodated state (AC: 8G61), using cryogenic electron microscopy (cryo-EM)(1). These structures are available from PDB; however, eIF5A1 is absent in the CR state. Therefore, the binding sites of eIF5A1 to RNAs and proteins were analyzed using IC, GA, and AC states. In each structure, eIF5A1 binds to two RNAs and two large ribosomal subunit proteins (LSUs): near Lys^50^ of eIF5A1 to 28S rRNA, near Lys^39^ and Arg^86^ to tRNA, at the N-terminus to RPL36A, and at the C-terminus to RPL10A protein (Table S4). Using this binding information, we attempted to illustrate the differences between eIF5A1 and eIF5A2. The amino acid sequences of eIF5A1 and eIF5A2 differ by 28 residues, 22 of which are at the C-terminus after Gln^90^. At the C-terminus of eIF5A1, which is bound to RPL10A via hydrogen bonding, hydrophobic interactions, or electrostatic interactions. Therefore, the structure of eIF5A2 was created using homology modeling, and MD simulations were carried out on the three-dimensional structures of eIF5A1-RPL10A and eIF5A2-RPL10A.

**Figure S12. The difference in the amino acid sequence at the C-terminus of eIF5A2 provides a dynamic structure that is distinct from that of eIF5A1.**

Three-dimensional structure of eIF5As interacting with the RPL10A protein. eIF5As and RPL10A are shown in green and cyan, respectively. Binding sites of eIF5As to RPL10A are shown in red. The structure of eIF5A2 was created using homology modeling, and MD simulation was performed.

**Figure S13. Schematic mechanisms through which eIF5A2 is regulated by polyamines at the translational level and is distinct from eIF5A1 in its participation in mRNA decoding.**

Polyamines stimulate eIF5A2 synthesis via suppression of miR-6514-5p function. Polyamines also alter the composition of ribosomes by modulating their protein expression levels. Alteration of ribosome composition facilitates the association of eIF5A2 and ribosomes; therefore, translation elongation of individual proteins regulated by eIF5A2 is distinct from that regulated by eIF5A1. The differences between the amino acids in eIF5A2 and eIF5A1 are shown in red.

**References**

1. Holm, M., Natchiar, S. K., Rundlet, E. J., Myasnikov, A. G., Watson, Z. L., Altman, R. B. *et al.* (2023) mRNA decoding in human is kinetically and structurally distinct from bacteria *Nature* **617**, 200-207
